# Yinzhihuang oral liquid in the treatment of neonatal jaundice: a meta-analysis

**DOI:** 10.1080/13880209.2016.1262432

**Published:** 2016-12-09

**Authors:** Jie Zeng, Su-jun Wang, Yong-mei Li, Hang-shan Li, Qian Luo, Yun-ying Huang, Qun Jiang, Li Wang

**Affiliations:** a Department of Pharmacy, The Fifth Affiliated Hospital of Guangzhou Medical University, Guangzhou, China;; b Department of Clinical Pharmacy, Guangdong Pharmaceutical University, Guangzhou, China

**Keywords:** Yinzhihuang oral liquid, neonatal jaundice, meta-analysis

## Abstract

**Context:** Yinzhihuang oral liquid, a well-known Chinese herbal formula, is a clinical drug for the treatment of neonatal jaundice, and a number of clinical trials have been published addressing this issue, but there is no comprehensive analysis that evaluates its efficacy for the treatment of newborn with hyperbilirubinaemia.

**Objective:** A meta-analysis was conducted to evaluate the efficacy of Yinzhihuang oral liquid on neonatal jaundice.

**Methods:** Search was performed throughout PubMed, Cochrane Library, EMBASE, Ovid, Wanfang, VIP Medicine Information System (VMIS) and China National Knowledge Infrastructure (CNKI) databases up to December 2015. The search terms were (Yinzhihuang oral liquid or Yinzhihuang oral solution), (neonatal jaundice or neonatal hyperbilirubinaemia), and (efficacy). Review Manager 5.2 software was used for analyzing the data. Data were pooled by using the random-effects models and expressed as relative ratio (RR), standardized mean difference (SMD) or mean difference (MD) with a 95% confidence interval (CI). The Cochrane tool was applied to assess the risk of bias of the trials.

**Results:** Yinzhihuang oral liquid in conjunction with other therapy increased effective rate of neonatal jaundice therapy (RR =1.14, 95%CI: 1.08–1.20). Yinzhihuang oral liquid significantly eliminated overproduced bilirubin which was measured by TSB or TCB at the third day and fifth day during the treatment {[third day, SMD = −1.63, 95%CI: −2.20 to (−1.06)], [fifth day, SMD = −5.00, 95%CI: −7.88 to (−2.12)]}; Yinzhihuang oral liquid significantly shortened jaundice subsiding time [MD = −3.20, 95%CI: −6.01to (−0.39)].

**Conclusion:** Yinzhihuang oral liquid can be considered as an effective treatment option for neonatal jaundice.

## Introduction

Many newborn babies develop jaundice in the first week of life. About 60% of term and 80% of preterm babies develop jaundice, and about 10% of breast fed babies are still jaundiced at 1 month (Rennie et al. 2010). Neonatal jaundice is caused by the unconjugated bilirubin imbalance between the production and elimination (Dennery et al. 2001). Generally, neonatal jaundice is harmless, but high concentration of unconjugated bilirubin level can develop acute encephalopathy of kernicterus (Brites 2012; Hussain et al. 2010; Sgro et al. 2006). The more serious consequences are permanent sequelas, such as deafness, cerebral palsy, dental dysplasia, variable intellectual disability and other neurologic damage among survivors (Gamaleldin et al. 2011; Mwaniki et al. 2012). Therefore, attention should be given to neonatal jaundice.

The clinical intervention on jaundice includes phototherapy, exchange transfusion and phenobarbitone. In the 1950s, exchange transfusion was the primary and successful form of therapy for severe neonatal jaundice (Abu-Ekteish et al. 2000; Johnson et al. 2002; American Academy of Pediatrics Subcommittee on Hyperbilirubinemia 2004; Bhutani et al. 2004; Mishra et al. 2008). The mortality rate of exchange transfusion was higher and higher from 1960s to 1980s, and the mortality rate varied from 0.65% to 3.2%. Cause of death ascribed to exchange transfusion included bacterial sepsis, nutrient loss, enterocolitis, pneumonia and cardiovascular collapse (Panagopoulos et al. 1969; Kitchen 1970; Dikshit & Gupta 1989; Guaran et al. 1992). In the late 1960s, phototherapy became widespread in the United States (Bryla 1985). The mechanism of phototherapy is that absorption of light through the skin converts unconjugated bilirubin into water-soluble bilirubin isomer, which is excreted from the stool and urine. However, there also have some side-effects should be aware to phototherapy, for instance, watery diarrhoea, water loss, skin rashes, blue baby syndrome and transient bronzing of the skin.

Currently, phenobarbitone is the main drug for treating neonatal jaundice (Levin et al. 1970). Trolle (1968) showed the ability of phenobarbitone to decrease the surum-bilirubin concentration in newborns. The decrease in serum-bilirubin concentration is most probably due to induction of enzymes in liver microsomes by phenobarbitone. The possible adverse effects of phenobarbitone on neonate have been reviewed by some researches. Phenobarbitone may increase enzyme activity of liver cells and that will accelerate the metabolism of certain drugs leading to less effect (Burns 1964; Fouts 1964; Conney 1967). Therefore, it is necessary to find another way to control bilirubin within normal range whilst avoiding the adverse effects of therapy.

Along with the development of Chinese medical science, traditional Chinese medicine has been widely used in healthcare in China. Chinese herbal formulas are comprised of multiple components and perform more efficiently. Chinese herbal formulas have been attracting increasing attention as their complementary therapeutic effects compared with western medicines (Normile 2003; Xue & Roy 2003). Yinzhihuang oral liquid, a Chinese herbal preparation, is officially listed in the Chinese Pharmacopoeia (China Pharmacopoeia Committee, 2010), and its main ingredients include *Artemisia capillaris* Thunb, *Gardenia jasminoides* J.Ellis, *Scutellaria baicalensis* Georgi and *Lonicera japonica* Thunb. Recently, in China, Yinzhihuang oral liquid has been reported to treat neonatal jaundice in many clinical trials (Qian et al. 2014; Wang & Su 2015; Yan & Ye 2014). These individual studies suggest that Yinzhihuang oral liquid have efficacy for the treatment of neonatal jaundice, but there is no meta-analysis to verify the efficacy of Yinzhihuang oral liquid. Therefore, we conducted a meta-analysis of clinical controlled trials to assess the therapeutic value of Yinzhihuang oral liquid for the treatment of neonatal jaundice.

## Materials and methods

### Search strategy

Comprehensive search of English and Chinese databases were performed by two researchers (JZ and SJW). Published studies were identified by searching PubMed, Cochrane Library, EMBASE, Ovid, Wanfang, VIP Medicine Information System (VMIS) and China National Knowledge Infrastructure (CNKI) databases up to 31 December 2015, with the search terms ‘Yinzhihuang oral liquid’, ‘Yinzhihuang oral solution’, ‘neonatal jaundice’, ‘neonatal hyperbilirubinemia’ and ‘efficacy’. Eligible studies were randomized trials that assessed the clinical efficacy of Yinzhihuang oral liquid for neonatal jaundice treatment in comparison with other routine treatment. All enrolment studies were approved by the authors.

### Inclusion criteria

Studies were included in the meta-analysis if they met the following criteria: (a) the study was randomized; (b) patients were neonate, with a confirmed diagnosis of jaundice; (c) Yinzhihuang oral solution was used alone or in combination with routine therapy, compared with the routine therapy as a control. Routine therapies include phototherapy, phenobarbitone, albumin injection or immune globulin injection; (d) outcome measures included one or more of the following indices: total efficacy rate, total serum bilirubin (TSB), transcutaneous bilirubin (TCB) and jaundice subsiding time.

### Exclusion criteria

The exclusion criteria were (1) reviews, nonclinical studies and case observations; (2) not RCTs; (3) reduplicated studies; (4) control groups received the intervention that treatment groups did not receive; (5) improper outcome measures; (6) meta-analysis, case reports, editorials and meeting abstracts.

### Data extraction

The following data were extracted from each study: first author, year of publication, number of patients, sex of patients, interventions in the treatment and control groups and outcomes.

### Quality assessment

The Cochrane risk of bias tool was used to assess the methodological quality of included RCTs. We assessed the quality of every published randomized trial on the basis of six elements: selection bias (random sequence generation and allocation concealment), performance bias, detection bias, attrition bias, reporting bias and others bias. The judgment was marked as ‘high risk’, ‘unclear risk’ or ‘low risk’.

### Statistical analysis

The Cochrane Review Manager 5.2 (Copenhagen, The Nordic Cochrane Center, The Cochrane Collaboration, 2012) was performed for the meta-analysis. Risk ratio (relative risk (RR)) was calculated for dichotomous outcome (e.g., efficacy rate). The standardized mean difference (SMD; difference in mean effects between groups divided by the pooled SD) and mean difference (MD) were calculated for continuous data. The *χ*^2^ test and the inconsistency index statistic (*I*^2^) for heterogeneity were conducted. If substantial heterogeneity occurred (*I*^2 ^>50% or *p* < 0.05), a random effect model was used to calculate the pooled RR, SMD or MD. If there was no observed heterogeneity, the fixed effect model will be chosen.

## Results

### Characteristics of included studies

**Figure 1.**
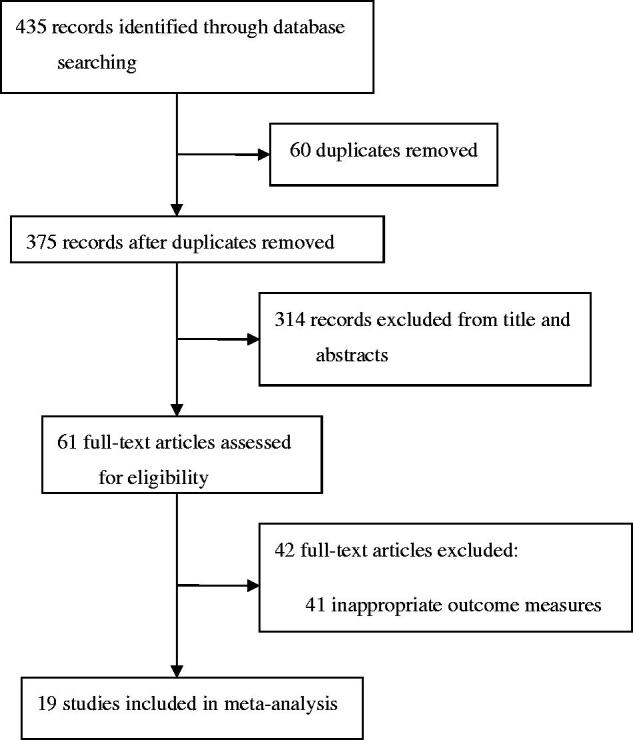
Study selection.

Figure 1 shows the process of study selection. 61 studies met the inclusion criteria according to information in the title and abstract, of which 41 were excluded, and the remaining 19 articles underwent full-text screening.

In total, 19 articles involving 2594 newborn babies with jaundice (1307 cases in the trial group and 1287 cases in the control group) were undergone meta-analysis. The basic characteristics of the included studies are listed in Tables 1 and 2.

**Table 1.** The characteristics of the included studies.

Author	Year published	Cased T/C	Sex male/female
Liang (2014)	2014	60/60	68/52
Lin and Cui (2011)	2011	64/64	76/68
Wang et al. (2010)	2010	90/90	100/80
Gao (2011)	2011	38/38	/
Shao and Li (2013)	2013	80/80	/
Chen (2013)	2013	77/83	95/65
Zhao et al. (2014)	2014	41/40	48/33
Liu et al. (2013)	2013	50/50	52/48
Xv (2011)	2011	60/60	69/51
Yu et al. (2013)	2013	102/98	109/91
Xiang (2014)	2014	123/123	137/109
Zhang (2014)	2014	62/61	65/58
Mu et al. (2015)	2015	43/43	46/40
Chen and Yang (2013)	2013	39/30	39/30
Yang and Ren (2011)	2011	24/25	28/21
Jiang (2011)	2011	150/150	174/126
Qiu and Wang (2014)	2014	60/60	/
Zhu (2014)	2014	40/40	46/34
Guo (2013)	2013	98/98	136/60

T: trial group; C: control group; /: no report.

**Table 2.** Intervention and outcome measures of the included studies.

	Intervention		
Author (year)	Trial group	Control group	Outcomemeasures
Liang (2014)	Phototherapy + YOL	Phototherapy	Efficacy
Lin and Cui (2011)	RT + YOL	RT	Efficacy
Wang et al (2010)	RT + YOL	RT	Efficacy
Gao (2011)	RT + YOL	RT	Efficacy
Shao and Li (2013)	Phototherapy + YOL	Phototherapy	Efficacy
Chen (2013)	RT + YOL	RT	Efficacy, TCB
Zhao et al. (2014)	RT + YOL	RT	Efficacy
Liu et al. (2013)	RT + YOL	RT	TSB
Xv (2011)	RT + YOL	RT	Efficacy
Yu et al. (2013)	RT + YOL	RT	Efficacy, TSB
Xiang (2014)	RT + YOL	RT	Efficacy
Zhang (2014)	RT + YOL	RT	Efficacy
Mu et al. (2015)	Phototherapy + YOL	Phototherapy	Efficacy
Chen and Yang (2013)	RT + YOL	RT	TSB
Yang and Ren (2011)	RT + YOL	RT	Efficacy
Jiang (2011)	RT + YOL	RT	Efficacy, TCB
Qiu and Wang (2014)	Phototherapy + YOL	Phototherapy	Efficacy
Zhu (2014)	RT + YOL	RT	Efficacy
Guo (2013)	RT + YOL	RT	Efficacy

YOL: Yingzhihuang oral liquid; RT: Routine treatment; TSB: total serum bilirubin; TCB: transcutaneous bilirubin.

### The quality assessment

**Figure 2.**
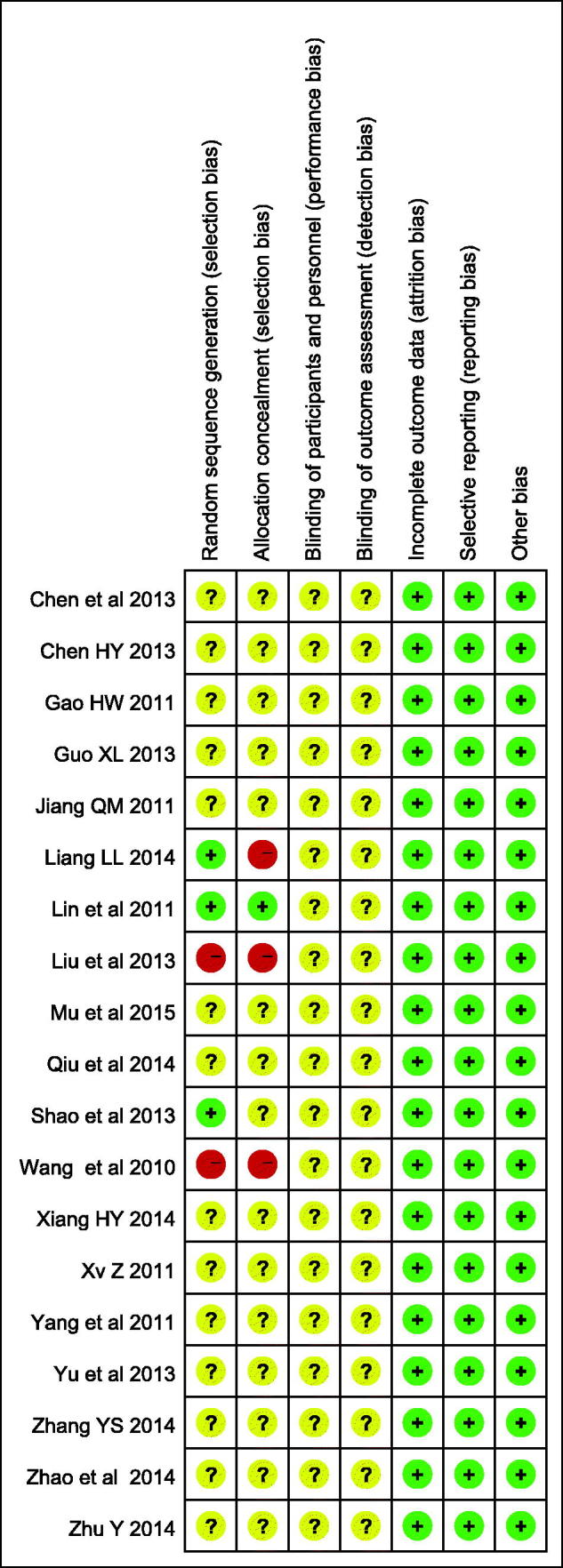
Methodological quality assessment of the risk of bias for each included study.

All the included RCTs were assessed to be low methodological quality. 19 articles used the random sequence generation method. One study (Liang 2014) used the ballot. Two articles used the numbers method (Lin & Cui 2011; Shao & Li 2013). The other two studies used the hospital order (Liu et al. 2013; Wang et al. 2010). The remaining others did not provide any detailed information. Therefore, we were unable to assess the quality of the random sequence generation methods. Allocation concealment was mentioned in four of the studies (Wang et al. 2010; Lin & Cui 2011; Liu et al. 2013; Liang 2014). The blinding of participants and personnel and blinding of outcome assessment blinding were not conducted in any trial. No articles had incomplete outcome data and selective reporting (Figure 2).

### Efficacy of Yingzhihuang oral liquid on neonatal jaundice

**Figure 3.**
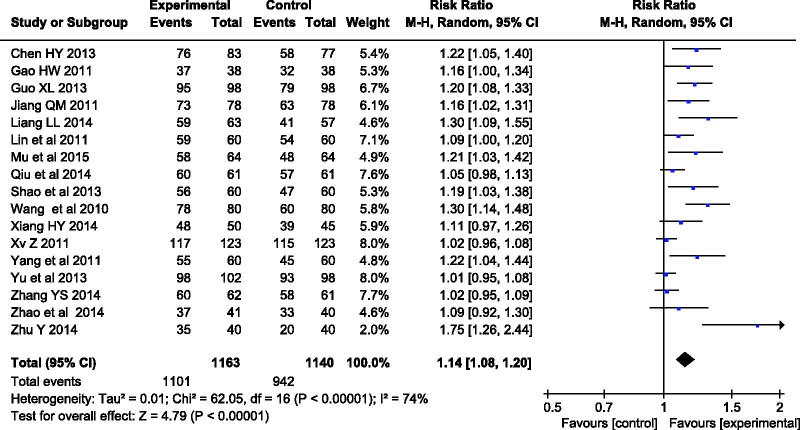
Effective rates of comparison between treatment and the control group.

The nineteen included studies compared the clinical efficacy rate between the treatment and control groups. Three classes were used to evaluate the effects of treatment: significant efficacy, efficacy and inefficacy. The total efficacy rate was the combination of the significant efficacy and efficacy rate. Heterogeneity occurred in efficacy rate of treatment group between control group (*χ*^2 ^=^ ^62.05, *p* < 0.00001, *I*^2 ^=^ ^74%). Therefore, a random-effect model was adopted for statistical analysis, and the total efficacy rate favoured the treatment group over the control group, with a statistically significant difference (RR = 1.14, 95%CI: 1.08–1.20, *p* < 0.00001), and the results are outlined in Figure 3.

### Biochemical indicators

**Figure 4.**
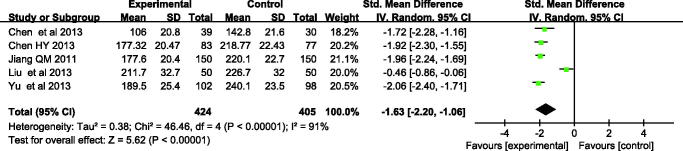
Meta-analysis of the bilirubin levels of Yingzhihuang oral liquid group and the control group during treating neonatal jaundice on third day.

**Figure 5.**
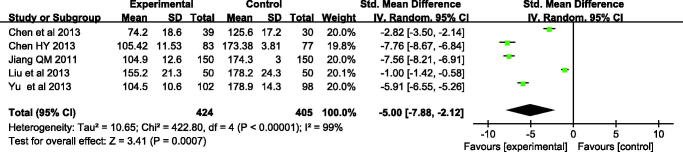
Meta-analysis of the bilirubin levels of Yingzhihuang oral liquid group and the control group during treating neonatal jaundice on fifth day.

In this analysis, three trials reported the total serum bilirubin levels on the third day and fifth day during treatment, and another two studies reported the transcutaneous bilirubin. At the third day, according to *χ*^2^and *I*^2^ analysis, heterogeneity was observed between two groups [(*χ*^2 ^=^ ^46.46, *p* < 0.00001, *I*^2 ^=^ ^91%)]. Therefore, the random-effect method was used to analyze the data. The bilirubin level at the third day was lower in the Yingzhihuang oral liquid group than in the control group, and the difference was statistically significant [SMD = −1.63 μmol/L, 95%CI: −2.20 to −1.06, *p* < 0.00001]. On the fifth day, there was significant difference between the two groups (SMD = −5.00 μmol/L, 95%CI = −7.88 to −2.12 μmol/L, *p* = 0.0007), with high heterogeneity among these studies (*I*^2 ^=^ ^99%, *p* < 0.00001). These results further supported that Yingzhihuang oral liquid group alleviated neonatal jaundice than the control group (Figures 4 and 5).

### Jaundice subsiding time

**Figure 6.**
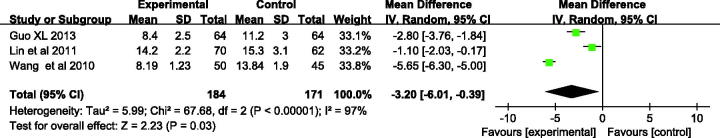
Meta-analysis of the subsiding time of jaundice of Yingzhihuang oral liquid and the control group.

Three studies reported the subsiding time of jaundice. The random-effect model was used because of the obvious heterogeneity across the trials included in the meta-analysis (*p* < 0.00001, *I*^2 ^=^ ^97%). The MD was −3.20 (*p* = 0.03) with a 95%CI of −6.01 to −0.39. Therefore, Yingzhihuang oral liquid group and control group showed significant differences in their ability to reduce the serum bilirubin concentration in patients (Figure 6).

## Discussion

Jaundice is one of the most common causes of morbidity in newborns. Yingzhihuang oral liquid is a traditional Chinese medicine, which is widely used to treat neonatal jaundice in clinical practice. This meta-analysis included 19 RCTs involving 2594 participants. There were 1307 patients in Yingzhihuang oral liquid group and 1287 patients in the control group. Our results indicated that compared with control group, Yingzhihuang oral liquid treatment group achieved a higher efficacy rate of therapy. Moreover, Yingzhihuang oral liquid treatment group significantly reduced the bilirubin level vs. the control group at treatment period. Furthermore, treatment group significantly shortened the subsiding time of jaundice.
